# Autologous fecal microbiota capsules are safe and potentially preserve beta-cell function in individuals with type 1 diabetes

**DOI:** 10.1080/19490976.2025.2563155

**Published:** 2025-10-05

**Authors:** Pleun de Groen, Coco M. Fuhri Snethlage, Koen Wortelboer, Sevilay Tokgöz, Mark Davids, Xanthe Verdoes, Florine H.M. Westerbeke, Rick I. Meijer, Martin Gotthardt, Willem M. de Vos, Hilde Herrema, Max Nieuwdorp, Nordin M.J. Hanssen

**Affiliations:** a Department of Internal and Vascular Medicine, Amsterdam UMC, University of Amsterdam, Amsterdam, The Netherlands; b Department of Medical Imaging, Nuclear Medicine, Radboud University Medical Center, Nijmegen, The Netherlands; c Department of Internal Medicine, Radboud University Medical Center, Nijmegen, The Netherlands; d Laboratory of Microbiology, Wageningen University, Wageningen, The Netherlands and Human Microbiome Research Program, University of Helsinki, Helsinki, Finland; e Amsterdam Diabeter Center, Amsterdam, The Netherlands

**Keywords:** Type 1 diabetes, fecal microbiota transplantation, autologous fecal microbiota capsules, beta-cell function

## Abstract

This study investigated the safety and feasibility of daily ingestion of autologous lyophilized fecal microbiota capsules (a-LFMCs) for preserving beta-cell function in individuals with type 1 diabetes (T1D). We evaluated a-LFMC in an open-label, single-arm pilot study (NCT05323162) with 10 individuals with T1D. The study included a 3-month run-in period, 3 months of daily a-LFMC treatment, and a 3-month follow-up. Beta-cell function was assessed using mixed-meal stimulated C-peptide area under the curve (AUC). During the run-in period, beta-cell function significantly declined (mean ΔAUC −12.02 ± 5.09 nmol/L*min, *p* = 0.025). There was no decrease in beta-cell function during the a-LFMC treatment period (mean ΔAUC 0.76 ± 5.09 nmol/L*min, *p* = 0.88) and the follow-up period (mean ΔAUC 0.96 ± 5.09 nmol/L*min, *p* = 0.85). No serious adverse events occurred, though constipation increased during the treatment period (0% vs. 30%, *p* = 0.021). a-LFMC treatment was found to be safe and potentially contributes to preserving beta-cell function in T1D patients. A larger randomized placebo-controlled trial is needed to confirm these promising findings.

## HIGHLIGHTS

• Lyophilized fecal microbiota capsules (LFMCs) were found to be safe and were well-tolerated by participants with T1D.
• C-peptide levels did not decline during the 3-month LFMC treatment or the 3-month follow-up.
• Indigestion, abdominal pain, gastro-oesophageal reflux disease, and diarrhoea did not differ between the run-in and treatment period.
• Constipation increased during the treatment period but was resolved in the follow-up period

## Graphical abstract

**Figure:**
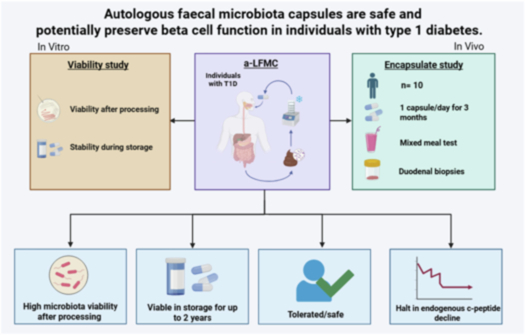

## Introduction

Type 1 diabetes (T1D) is a chronic T-cell-mediated autoimmune disease characterized by insulitis leading to destruction of the pancreatic beta cells, resulting in the progressive loss of insulin-secreting capacity.^1–3^ T1D pathophysiology is heterogeneous, and both disease onset and progression are attributed to a complex interplay between genetic and environmental factors.^3–5^ Disease incidence is rapidly increasing in individuals without at-risk human leukocyte antigens (HLA) types, which is attributed to increased environmental pressure.^6–8^ Maintenance of residual beta-cell function is a major prognostic factor for better outcomes in T1D patients and is associated with better glycemic control and a lower risk of hypoglycemia.^9^^,^^10^ Currently, there is no cure for T1D, and efforts to halt disease progression have shown limited success, with most resulting in delay of beta-cell degeneration.^11–14^

Perturbations of the gut microbiome have been associated with all stages of T1D development.^15–21^ Multiple studies have shown that the microbiome of individuals with T1D differs from that of healthy controls. While some early studies reported a decrease in microbiome diversity prior to auto-antibody seroconversion, larger and more recent prospective studies have not consistently observed significant differences in diversity prior to seroconversion near or after T1D diagnosis.^21^^,^^22^ Similarly, it has been shown that children with T1D who maintain beta-cell function throughout the disease have a different microbiome than those who no longer secrete C-peptide.^19^

A recent study showed that altering the intestinal microbiome through autologous fecal microbiota transplantation (FMT) via a duodenal tube may halt disease progression in new-onset T1D.^23^ It is thought that the homeostasis between the duodenal microbiome and the innate immune system is disrupted in T1D, resulting in the generation of auto-reactive CD8 + T-cells.^24^ The HLA type influences the baseline microbial composition and risk of autoimmune disease, though whether the HLA type affects microbial strain engraftment remains a topic of emerging research.^25^ Although strain engraftment of microbiota may be influenced by host factors, a favorable response to autologous FMT is also plausible because the colon harbors the greatest microbial load and diversity in the body. Moreover, the gastrointestinal tract is the primary site where tolerogenic immune responses to the commensal microbiota are induced.^26^^,​​^​​​​​^27^ Indeed, germ-free non-obese diabetic (NOD) mice that lack normal coprophagy of their fecal microbiome develop more insulitis.​​​​​^28^

FMT is commonly performed via nasoduodenal tubes or colonoscopy, and the procedure is considered cumbersome for the recipient and labor intensive. However, as FMT likely has to be repeated periodically for a prolonged effects in T1D patients, these traditional invasive administration methods are unsuitable. Interventions with probiotics, single microbes, or metabolites have thus far achieved limited success, partly because no consensus has been reached on which specific microbes are involved in type 1 diabetes (T1D).^29^ Nonetheless, several recent studies have shown that metabolite-based therapies can effectively modulate immune markers and inflammatory responses in T1D and related conditions.^30^^,^^31^ The aim of this study was to test the safety and efficacy of daily ingested autologous lyophilized fecal microbiota capsules (a-LFMCs) to preserve beta-cell function in individuals diagnosed with T1D between 0.5 and 3.5 years ago (The ENCAPSULATE-DM1 study).

## Research design and methods

### Study participation and design

**Figure 1.**
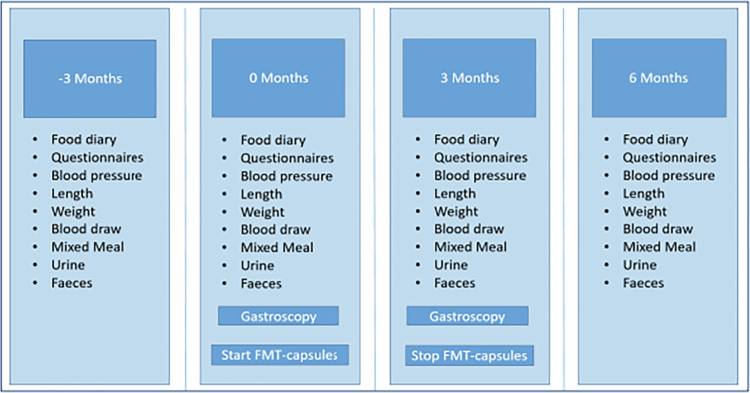
Study overview. Study visits are performed at −3, 0, 3 and 6 months.

This open-label, single-arm pilot study recruited 10 participants with recently diagnosed T1D. We opted to include individuals with 0.5–3.5 y of diabetes duration because the inflection point for loss of residual beta-cell function stabilizes at approximately 7–8 y, as found in at least two separate source populations,^32^ including our own.^33^ Based on the spline graphs of these papers, we argue that including individuals with diabetes durations between 0.5 and 3.5 y would yield the steepest decline in beta-cell function. The participants were screened for eligibility through the GUTDM1 study (NL-OMON49369, METC_2020_105), a cross-sectional cohort that focuses on the role of environmental factors in preserving beta-cell function in T1D.​​​​​​^9^^,^^34^ The study visits took place between May 2022 and June 2024 at the Amsterdam University Medical Centre, which is located at the Academic Medical Centre (AMC). This study included four visits in total (Figure 1). To assess the natural decline in insulin secretory capacity, all individuals participated in a 3-month run-in phase prior to the intervention (*t* = −3 months to 0 months). During this period, no treatment was administered. Insulin secretory capacity was measured at the start and end of the run-in phase. The daily ingestion of a-LFMCs for three months commenced only after this run-in phase, marking the start of the treatment period (*t* = 0 to *t* = 3 months). Before and after intervention with a-LFMCs, duodenal biopsies were collected via gastroduodenoscopy after an overnight fast, with the last a-LFMCs taken 24 h prior. This was followed by three months of follow-up (*t* = 3 to *t* = 6 months), before and after which insulin secretory capacity was measured. The study was approved by the IRB of Amsterdam UMC (NL-OMON50429, clinicaltrail.gov ID: NCT05323162).

### In and exclusion criteria

**Figure 2.**
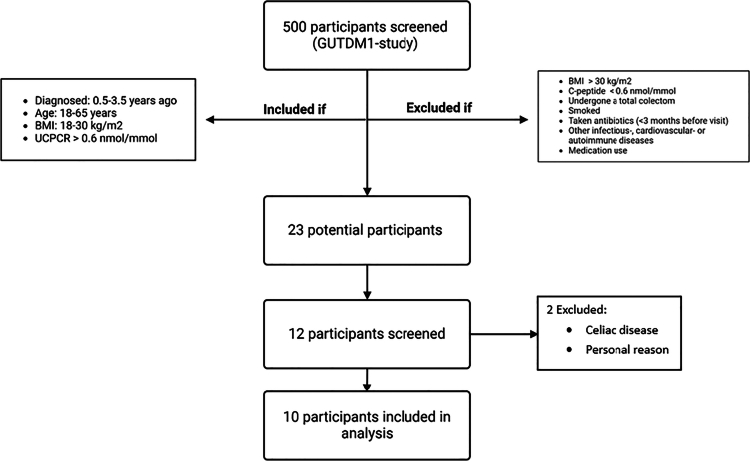
Flowchart of in and exclusion.

To be included, participants had to be >18 y of age and diagnosed with insulin-dependent auto-antibody positive T1D in accordance with the ADA/EASD guidelines within the last 0.5−3.5 y and they had to have detectible plasma (>0.05 nmol/L) or urinary C-peptide (UCPRC >0.01 nmol/mmol).^35^ They had to be willing to provide written informed consent in adherence with the Declaration of Helsinki before enrolment. Patients were excluded if their BMI exceeded 18−30 kg/m^2^; if they had undergone a total colectomy, smoked, had recently taken antibiotics (<3 months before visit), had other infectious, cardiovascular, gastrointestinal or autoimmune diseases; or were on any type of medication (Figure 2).

### Processing of the autologous lyophilized fecal matter

**Figure 3.**
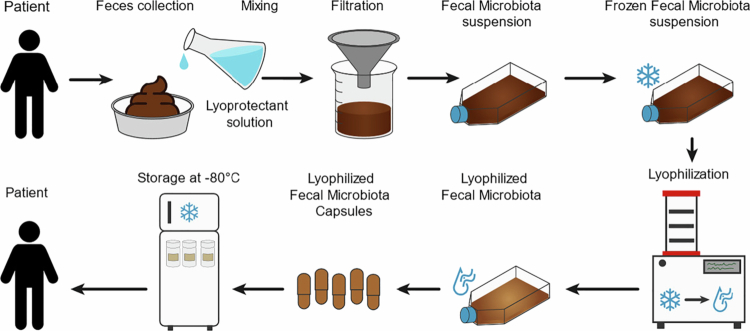
Lyophilized autologous fecal microbiota capsules production process from received feces to capsule consumption.

All steps of capsule production are displayed in Figure 3 and are performed aerobically. After the 3-month run-in phase of the study, the participants were treated on a daily basis with one capsule containing autologous lyophilized fecal matter for a period of 3 months. As feces are required for the manufacture of the a-LFMCs, participants were instructed to collect at least 150 g of their own fresh feces at timepoint −3 months. If less than 150 g of stool was delivered, participants were asked to collect additional stool until a sufficient amount was collected to produce enough a-LFMCs. The feces were delivered refrigerated (2−8 degrees) within 2 h to the investigator at the AMC.

The autologous feces were processed directly by homogenizing the feces 1:1 with a lyoprotectant solution (10% trehalose and 5% maltodextrin in sterile saline, all pharmaceutical grade). The fecal suspension was then filtered through double non-woven sterile gauzes in a sterile metal funnel to remove any particulate matter. The resulting fecal microbiota suspension was transferred to sterile flasks (50 ml/flask) and frozen at −80 °C. Prior to the start of the treatment at 0 months, but at least after an overnight freeze, the flasks of frozen fecal microbiota were placed in the drying chamber of a freeze dryer (Zirbus VaCo_2_) and lyophilized for 48 h. The resulting lyophilised fecal microbiota were transferred to a mortar, ground to a fine powder, and mixed with a lubricant (pharmaceutical grade silica) to improve the flowing properties of the powder. If necessary, filler (maltodextrin, pharmaceutical grade) was added to the powder mixture to achieve the required volume for the number of a-LFMCs to be filled.

Finally, the powder mixture was encapsulated in capsules with intrinsic enteric properties (Vcaps Enteric®, Capsugel). The a-LFMCs were dispensed into opaque 100 ml high-density polyethylene containers (DUMA Twist-off), with 30 a-LFMCs per container, including a silica canister. The containers were labeled in accordance with GMP Annex 13 and stored in a freezer at −80 °C. The participants stored the capsules at home in the freezer until ingestion. They were instructed to ingest 1 capsule a day with a glass of water on an empty stomach in the morning, preferably an hour before breakfast (while still fasted). In total, the participants ingested a set dose of 90 capsules, which represented 150 g of stool. At the end of the three-month intervention period, any remaining a-LFMCs were returned by the study subjects to the investigator, were counted and destroyed by the study pharmacist.

### Microbial viability during processing and stability during storage

Viability of the microbiota was measured using a validated plate counting technique.^36^^,^^37^ To test for microbiota viability during the preparation process (as previously described, see Figure 3), samples were collected at various stages of the process. These included fresh fecal samples; fresh, frozen, and lyophilized fecal microbiota suspension samples; and samples of the capsules containing the lyophilized fecal microbiota. After resuspension and dilution, the samples were plated on blood agar plates and incubated for 48 h under aerobic and anaerobic conditions. Using the plate counting technique, the number of viable aerobic and anaerobic bacteria present were determined, expressed as colony-forming units CFU. To determine the stability of the capsules, they were stored at various temperatures (room temperature, 4 and −80 °C) for a period of 2 y. The viability of the fecal microbiota in the capsules was again determined using the plate counting technique over time.

### Determination of the fecal microbiome and small intestinal microbiota composition

Prior to (0 months) and after (3 months) treatment, participants underwent a gastroscopy to collect duodenal biopsies. In addition, a quality control sample of the capsule was taken. The DNA extraction from both duodenal biopsies and capsule contents was performed using a repeated bead-beating protocol, and the purification of the lysates was done using the Maxwell® system (RSC Promega, Cat# AS4500).

For both sample types, 300 µL S.T.A.R. buffer (Roche Diagnostics, Cat# 03335208001) was added to the bead-beating tube. For duodenal biopsies, proteinase K was included, and the samples were incubated at 56 °C for 60 min while shaking (1000 rpm). After incubation, both sample types were mechanically disrupted using the bead beater (Bertin precyllus 24, Cat# P000669-PR240-A) (5.5 ms, 3 × 1 min). After homogenization, the samples were incubated at 95 °C for 10 min while shaking (1000 rpm). After incubation, the samples were centrifuged at 4 °C for 5 min at full speed (14,000 rpm). The supernatant was transferred to a nuclease-free tube. The bead-beat tube was kept, and 150 µL of S.T.A.R. buffer was added. The bead beating and centrifugation steps were repeated. The supernatant was transferred and homogenized with the supernatant of the first step to an existing nuclease-free tube. The DNA was purified on the Maxwell® (RSC Promega, Cat# AS4500), using the Maxwell® RSC Blood DNA kit (Cat# AS1400) according to the manufacturer’s instructions. After isolation, 16S rRNA sequencing was performed by the Microbiome Centre Amsterdam, as previously described.^23^ Amplification of the 16S rRNA gene V3–V4 region was performed by one step Polymerase Chain Reaction (PCR) using primers composed of the Illumina adapter sequence, a unique barcode for sample identification, padding/linker sequences, and region-specific primers targeting the V3–V4 region. The forward primer (P5) sequence was AATGATACGGCGACCACCGAGATCTACACNNNNNNNNTATGGTA ATTGGCCTACGGGAGGCAGCAG, and the reverse primer (P7) sequence was CAAGCAGAAGACGGCATACGAGATNNNNNNNNAGTCAGTCAGCCGACTACHVG GTATCTAATCC. The purified PCR product was quantified using the Qubit® dsDNA BR kit (Thermo Fisher, Cat# Q32853) and sequenced on the Illumina MiSeq platform.

The amplicon sequences were analysed using a vsearch (v2.15.2) based pipeline.^38^ Paired-end reads were merged, with a maximum difference of 100 bases permitted and staggered overlap allowed. The ASVs were inferred from the reads with an expected error rate of less than 1.5 using the cluster_unoise with centroids algorithm, with a minimum size of 4. The chimaeras were subsequently removed using the uchime3 de novo method. For each sample, the abundance of ASVs was determined by mapping the merged reads against ASV sequences using the usearch_global algorithm with a 0.97 distance cutoff. Taxonomy was assigned using R (v4.4.3) and the dada2 assign taxonomy function, utilizing the Silva (v132) reference database.^39^^,^^40^ A phylogenetic tree was generated using MAFFT (v7.310) and FastTree (v2.1.11).​​​​​^41^^,^^42^

### RNA extraction and cDNA synthesis for duodenal gene expression analysis

Fresh duodenal biopsy samples were snap-frozen immediately after collection and stored at −80 °C until processing. Total RNA was isolated using a combined TRIzol™ (Invitrogen™, Lot#23223901) and RNeasy MinElute Cleanup Kit (Qiagen, cat#74204) protocol to ensure that high-quality RNA was suitable for downstream qRT-PCR analysis. Each biopsy was transferred to a bead-beating tube containing 300 µL TRIzol™ reagent and homogenized for 30 s using a bead beater (Bertin precyllus 24, Cat# P000669-PR240-A). An additional 500 µL of TRIzol™ reagent was added, and the samples were incubated for 2 min at room temperature. The homogenate, along with 200 µL of chloroform, was transferred to a 1.5 mL tube, vigorously shaken, and incubated for 3 min at room temperature. Phase separation was performed by centrifugation at 12,000 × g for 15 min at 4 °C in a heavy phase lock gel tube (QuantaBio, cat#2302830, lot#55705818). The aqueous phase was transferred to a new tube, mixed 1:1 with 70% ethanol, and applied to an RNeasy MinElute spin column. The column was washed sequentially with 500 µL of RPE buffer and 500 µL of 80% ethanol, with centrifugation steps at 8,000 × g (15 s for the RPE wash; 2 min for the ethanol wash) at 4 °C. After a final centrifugation at maximum speed for 5 min to dry the membrane, the RNA was eluted in 14 µL of RNase-free water by incubating the water on the column membrane for 1 min at room temperature followed by centrifugation at maximum speed for 5 min at 4 °C. The RNA concentration and purity were assessed using the synergy H1 plate reader (BioTek).

Approximately 1 µg of RNA was used for cDNA synthesis using the SensiFAST™ cDNA Synthesis Kit (Meridian, cat#BIO-65054, lot#RA653-B338060). The RNA samples were diluted to a final volume of 15 µL. The master mix was prepared on ice with 4 µL of 5 × TransAmp Buffer and 1 µL of reverse transcriptase per sample and added to the RNA. Reverse transcription was performed in a thermal cycler using the following program: 25 °C for 10 min (primer annealing), 42 °C for 15 min (reverse transcription), and 85 °C for 5 min (enzyme inactivation), followed by a hold at 4 °C. The samples were stored at −20°C until further use.

qPCR reactions were performed in a 384-well plate. For each reaction well, 2 µL of diluted cDNA (7.5 ng/µL) was used. A qPCR master mix was prepared for each primer pair, consisting of 2.5 µL SYBR Green and 0.5 µL 10 µM forward and reverse primers. qPCR was performed using a Bio-Rad CFX 384-well system (Supplementary Table 1). The thermal cycling protocol began with an initial denaturation step at 95 °C for 10 min, followed by 39 cycles, each consisting of denaturation at 95 °C for 15 s and combined annealing and extension at 60 °C for 30 s. Fluorescence was measured at the end of each annealing/extension step to monitor amplification in real time. After amplification, a melt curve analysis was conducted by increasing the temperature from 65 to 95 °C in increments of 0.5 °C, holding each step for 5 s, to verify product specificity and assess the presence of primer-dimers or non-specific amplification products. All data acquisition and analysis were carried out using the Bio-Rad CFX Manager software.

#### Data collection

During the study visits, participants were asked to complete standardized questionnaires on their general health and diabetes characteristics (i.e., duration of diabetes, medication use, type of RT-CGM/IS-CGM device and presence of neuropathy, retinopathy, nephropathy or cardiovascular disease, and daily insulin dose). The participants were required to complete food diaries for 3 d prior to their visit, to monitor the intake of dietary macronutrients (fibres, fat, proteins and carbohydrates).^43^ Furthermore, they were asked to collect 24-h urine samples and feces samples. During the visits, height, weight and blood pressure were measured. Gastro-intestinal symptoms were measured using the validated gastro-intestinal symptoms rating scale (GSRS).^44^^,^^45^ The GSRS is a questionnaire containing fifteen questions about the presence of gastrointestinal symptoms. The participants rated their gastrointestinal symptoms on a 7-point Likert scale, where "1" indicated no symptoms and "7" indicated very severe symptoms. The symptoms were categorized into five syndromes: gastroesophageal reflux disease (GERD), abdominal pain, diarrhea, constipation, and indigestion. Ratings above "3" were considered clinically relevant. If participants had an average score above "3" on questions related to a specific syndrome, they were classified as having that syndrome. Those without any syndrome were classified as not having gastrointestinal symptoms.

### HLA typing

DNA isolation and genotyping were performed using Immunochip as described earlier,^46^ and subsequently, the imputation for chromosome 6 (which contains the HLA region) was performed with the Michigan server, using four-digit multiethnic HLA reference panel v2 (hg19/GRCh37), Eagle v2.4.^47^ We identified HLA haplotypes DR3/3 (DR3/DQ2 homozygotes), DR3/4, DR4/4 (DR4/DQ8 homozygotes), and DR3 or DR4 heterozygotes, since these HLA haplotypes are frequently associated with T1D development.^48^^,^^49^ A DR3/DQ2 haplotype was defined as DRB1*03:01–DQA1*05:01–DQB1*02:01, and a DR4/DQ8 haplotype was defined as DRB1*04:01/DRB1*04:04–DQA1*03:01–DQB1*03:02. We divided the HLA genotypes into high-risk (DR3/3, DR3/4, or DR4/4), intermediate-risk (DR3/X or DR4/X) or low-risk (other) groups.

### Mixed meal tolerance tests and insulin secretion capacity

Mixed meal tolerance tests (MMT) were performed during all visits (timepoints −3, 0, 3 and 6 months). The participants fasted (>8 h) and used only 30% of their long-lasting exogenous insulin the night before starting the MMT. Fasted blood samples were collected for standard biochemistry (glucose, HbA1c, lipid profile, liver, and kidney function), plasma metabolomics, autoantibodies, HLA typing and RNA sequencing. EDTA plasma samples were sent to Nomic Bio for proteomic analysis (Supplementary Table 2). Boost high-protein nutritional drinks (Nestlé Nutrition, 89 Vervey, Switzerland) were used to perform the MMT, in line with previous T1D research.^23^ The participants drank 6 ml/kg of boost high protein with a maximum of 360 ml for each MMT and consumed no short-acting insulin until the end of the MMT. Blood was collected at timepoints 0, 15, 30, 45, 60, 90 and 120 min of MMT to determine stimulated C-peptide release. The detection limit for the C-peptide assay was <0.01 nmol/L. All blood samples were processed and stored at −80 °C until all participants had completed the study and analyzed together. Residual beta-cell function was determined as the stimulated C-peptide area under the curve (AUC) for 2 h (AUC0-120 min) after the start of the MMT and reported as time normalized to nmol/L as previously described.^50^^,^^51^

### Statistical analysis

Statistical analyses were performed using Rstudio 4.4.3 and GraphPad Prism 10. Data were presented as mean ± standard deviation or as the median with the interquartile range as appropriate for distribution. The normality of the variables was determined using histograms and QQ plots. Differences in C-peptide AUC were measured using both ANOVA and Students *t*-test as appropriate for all individual timepoints. The viability of the microbiota during processing of the LFMCs was tested for significance using a Wilcoxon matched pairs signed rank test. All microbiome analyses were performed with Bioconductor version 3.19 using the Phyloseq library (built under Rstudio version 4.4). Differences in alpha diversity between timepoints and the a-LFMCs were measured using Shannon index and by phylogeny through phylogenetic diversity. Bray–Curtis dissimilarity was used to measure beta diversity between timepoints and the a-LFMCs. Individual microbiota differences were displayed at the phylum level for all participants. All 16S microbiome analyses were adjusted for multiple testing using the Benjamini–Hochberg procedure to control the false discovery rate. A *p*-value of 0.05 after correction for multiple testing was deemed as statistically significant.

## Results

### Characteristics of the study population

Among the 10 participants included in this study, the median age was 24.0 [23.3–30.5], and 70% were male, with a median duration of T1D of 2.0 [1.0–3.0] years. At baseline, the median time in the range was 92.5% [82.0−97.3], the mean HbA1c was 44.5 ± 8.6 mmol/mol, and the mean fasting C-peptide was 0.21 [0.11−0.49] nmol/L (Table 1). At the time of inclusion, the anti-GAD and IA2 levels were 12.3 [2.0–71.0] U/mL and 174.0 [5.0–250.0] U/mL, respectively. The HLA types were 10% low risk, 30% intermediate risk and 40% high risk.

**Table 1.** Participant characteristics.

Characteristic	MMT1	MMT2	MMT3	MMT4	*p*
Participants (*n*)	10	10	10	10	
BMI (kg/m^2^)	25.3 (24.3- 27.8)	25.0 (24.1- 27.6)	25.0 (24.4- 27.7)	25.0 (24.2- 28.2)	0.984
Time in range (%)	92.5 (82.0- 97.3)	92.6 (70.0- 97.0)	87.0 (77.0- 95.6)	90.0 (73.0- 95.7)	0.914
Time above range (%)	7.0 (2.0- 15.8)	7.1 (2.5- 28.0)	9.0 (3.6- 22.0)	4.0 (3.0- 26.0)	0.980
Time below range (%)	1.0 (0.7- 1.0)	1.0 (0.4- 2.0)	2.0 (0.8- 4.0)	1.0 (0.7- 1.8)	0.540
Glucose CV (%)	26.2 ± 8.3	26.2 ± 6.2	29.0 ± 8.9	24.7 ± 6.8	0.734
Fasting C-peptide (%)	0.2 (0.1- 0.2)	0.1 (0.1- 0.2)	0.1 (0.1 - 0.3)	0.1 (0.1- 0.2)	0.799
HbA1c (mmol/mmol)	44.5 ± 8.6	47.9 ± 8.9	45.3 ± 9.0	46.8 ± 11.9	0.873
Fasting Glucose (mmol/L)	8.0 ± 5.0	6.5 ± 1.1	8.0 ± 4.0	8.3 ± 4.3	0.732
Daily insulin dose (U/day)	33.3 (17.3- 38.5)	33.8 (29.3- 42.3)	34.5 (25.4- 39.4)	36.3 (30.8- 46.9)	0.650
Total cholesterol (mmol/L)	4.1 ± 0.7	4.1 ± 0.8	4.0 ± 0.6	4.0 ± 0.6	0.966
LDL (mmol/L)	2.3 ± 0.7	2.3 ± 0.7	2.2 ± 0.6	2.2 ± 0.4	0.977
HDL (mmol/L)	1.6 ± 0.4	1.6 ± 0.5	1.6 ± 0.4	1.5 ± 0.3	0.822
Total energy intake (kJ/day)	8009.4 (6883.0 - 8863.3)	8294.1 (7782.4 - 9557.7)	7579.4 (6708.5 - 7959.1)	7694.1 (6481.2 - 10723.7)	0.705
Fibre intake (g/day)	30.2 (24.2 - 36.8)	25.7 (22.5 - 38.1)	25.7 (18.3 - 32.1)	30.4 (25.4 - 35.7)	0.771
Protein intake (g/day)	84.9 (66.3, 105.9)	99.8 (87.9 - 115.9)	75.5 (56.5 - 100.7)	79.7 (70.7 - 110.9)	0.298
Carbohydrate intake (g/day)	188.2 (166.0 - 204.2)	173.8 (129.8 - 193.9)	155.6 (134.2 - 206.3)	178.2 (143.6 - 205.8)	0.671
Fat intake (g/day)	69.6 (60.4 - 100.5)	95.0 (68.7, 142.0)	67.4 (60.5 - 89.2)	76.7 (69.8 - 124.8)	0.771
Sugar intake (g/day)	61.7 (49.0 - 71.7)	46.3 (35.0 - 56.8)	57.0 (37.8 - 61.7)	52.7 (38.6 - 59.3)	0.361

The presented values are the mean and standard deviation; median and interquartile range (Q1, Q3); or number and percentage, as appropriate. Missing values (Supplementary Table 4).

### Microbial viability during processing and stability during storage

**Figure 4.**
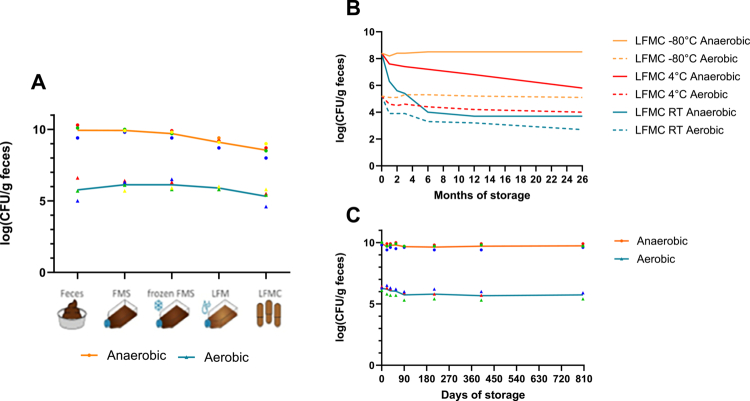
Viability and stability testing of the lyophilized fecal microbiota capsules LFMC. (a) The viability during LFMC processing from feces to fecal microbiota suspension (FMS), to frozen FMS, to lyophilized fecal microbiota (LFM), to LFMCs. (b) LFMC stability of both anaerobic and aerobic bacteria at room temperature (RT), 4 °C and −80 °C during 26 months of storage. (c) Stability of the frozen FMS stored at −80°C for 810 d.

The steps necessary to prepare the a-LFMCs are depicted in Figure 3. We tested microbial viability at different stages of the a-LFMC preparation using aerobic and anaerobic culturing techniques to determine the number of colony-forming units (CFUs). During the production of a-LFMCs, we observed a non-significant reduction in CFUs between the fecal microbiota suspension (FMS) and the a-LFMCs in both the anaerobic microbes (8.6*10^9^ ± 2.5*10^9^ vs. 4.8*10^8^ ± 4.0*10^8^ CFU/g feces, *p* = 0.13) and the aerobic microbes (1.6*10^6^ ± 0.9*10^6^ vs. 3.2*10^5^ ± 2.7*10^5^ CFU/g feces, *p* = 0.25) (Figure 4a). Next, we tested the stability of the a-LFMCs stored at different temperatures: room temperature, 4 and −80°C. Both the anaerobic and aerobic CFU’s within the a-LFMCs stored at −80 ℃ remained stable for over 2 y (anaerobes 2.9*10^8^ ± 1.8*10^8^ vs. 3.9*10^8^ ± 2.5*10^8^ CFU/g feces, *p* = 0.25; aerobes 2.0*10^5^ ± 1.4*10^5^ vs. 1.5*10^5^ ± 0.9*10^5^ CFU/g feces, *p* = 0.5; Figure 4b). In contrast, the a-LFMCs stored at 4 °C (anaerobes 2.9*10^8^ ± 1.8*10^8^ vs. 1.5*10^6^ ± 2.6*10^6^ CFU/g feces, *p* = 0.11; aerobes 2.0*10^5^ ± 1.4*10^5^ vs. 6.1*10^5^ ± 1.1*10^6^ CFU/g feces, *p* = 0.53; Figure 4b) and especially at room temperature (anaerobes 2.9*10^8^ ± 1.8*10^8^ vs. 6.3*10^4^ ± 1.1*10^5^ CFU/g feces, *p* = 0.11; aerobes 2.0*10^5^ ± 1.4*10^5^ vs. 5.7*10^2^ ± 7.3*10^1^ CFU/g feces, *p* = 0.13; Figure 4b) showed a significant decline in viable cells. Also the intermediate product, the frozen fecal microbiota suspension (FMS) remained stable when stored at −80 °C for over 2 y (anaerobes 5.4*10^9^ ± 2.7*10^9^ vs. 5.4*10^9^ ± 1.9*10^9^ CFU/g feces, *p* = 0.99; aerobes 2.0*10^6^ ± 1.2*10^6^ vs. 5.8*10^5^ ± 3.0*10^5^ CFU/g feces, *p* = 0.13; Figure 4c).

### Tolerance of a-LFMCs and changes in fasting plasma C-peptide and C-peptide AUC

The compliance, determined as the percentage of a-LFMCs daily ingested based on the number of leftover capsules, was 97.1% [93.3–100]. There were no serious adverse events reported in this study, and overall, the a-LFMCs were very well-tolerated. There was no significant difference in the presence of abdominal pain, GERD, indigestion or diarrhea across all timepoints, but there was an increase in constipation (0% vs. 30%, *p* = 0.021, Supplementary Table 3) during the treatment period, which was no longer present after the 3-month follow-up period.

**Figure 5.**
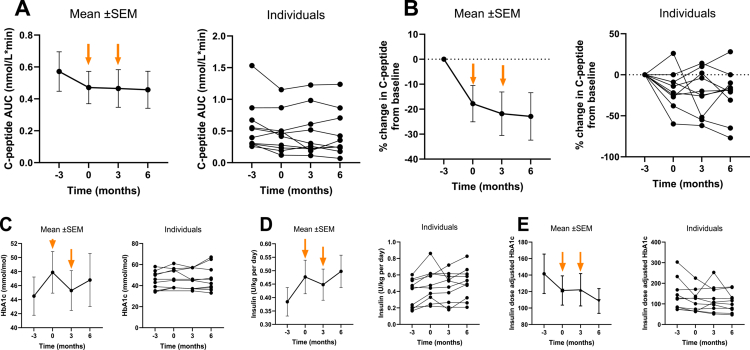
Overview of the mixed meal test results (mean and SEM). The time points included a 3-month run-in, followed by 3 months of lyophilized autologous fecal microbiota capsule treatment (orange arrows, timepoint 0–3 months) and a 3-month follow-up. (A) Mean/individual time normalized C-peptide AUC (nmol/L*min), (B) mean/individual change in C-peptide from baseline (%), (C) mean/individual HbA1c (mmol/mol), (D) mean/individual insulin dose (U/kg per day), and (E) mean/individual insulin dose adjusted for HbA1c.

There was a decline in the normalized C-peptide AUC in the run-in period before treatment (T = −3 to 0 months, mean ΔAUC, −1.01 ± 0.04 nmol/L*min, *p* = 0.047) (Figure 5a). In contrast, there was, on average, no decline in the C-peptide AUC during a-LFMC treatment period (T = 0−3 months, mean ΔAUC 0.01 ± 0.04  nmol/L*min, *p* = 0.88) and in the follow-up period (T = 3−6 months, mean ΔAUC 0.01 ± 0.03 nmol/L*min, *p* = 0.79). When investigating the relative difference in C-peptide AUC from baseline (T = −3 months), there was a significant decline from baseline C-peptide in the run-in period (T = −3 to 0 months, mean % difference, 17.80 ± 7.3  nmol/L*min, *p* = 0.037), but no difference in change from baseline in the treatment and follow-up period (Figure 5b).

HbA1c increased during the run-in period (T = −3 to 0 months, mean difference HbA1c in mmol/mol, 3.39 ± 1.01, *p* = 0.030), and stabilized after treatment and follow up (T = 0−3 months and T = 3−6 months) (Figure 5c). Similarly, the exogenous insulin dose (U/per day) increased during the run-in period (T = −3 to 0 months, mean difference in insulin dose 0.09 U/kg per day, *p* = 0.02), but this increase was halted during the treatment and follow-up period (Figure 5d). No significant changes in plasma cytokine concentrations were observed between pre- and post-intervention time points (data not shown).

### Changes in fecal and small intestinal microbiota and capsule/gut comparisons

**Figure 6.**
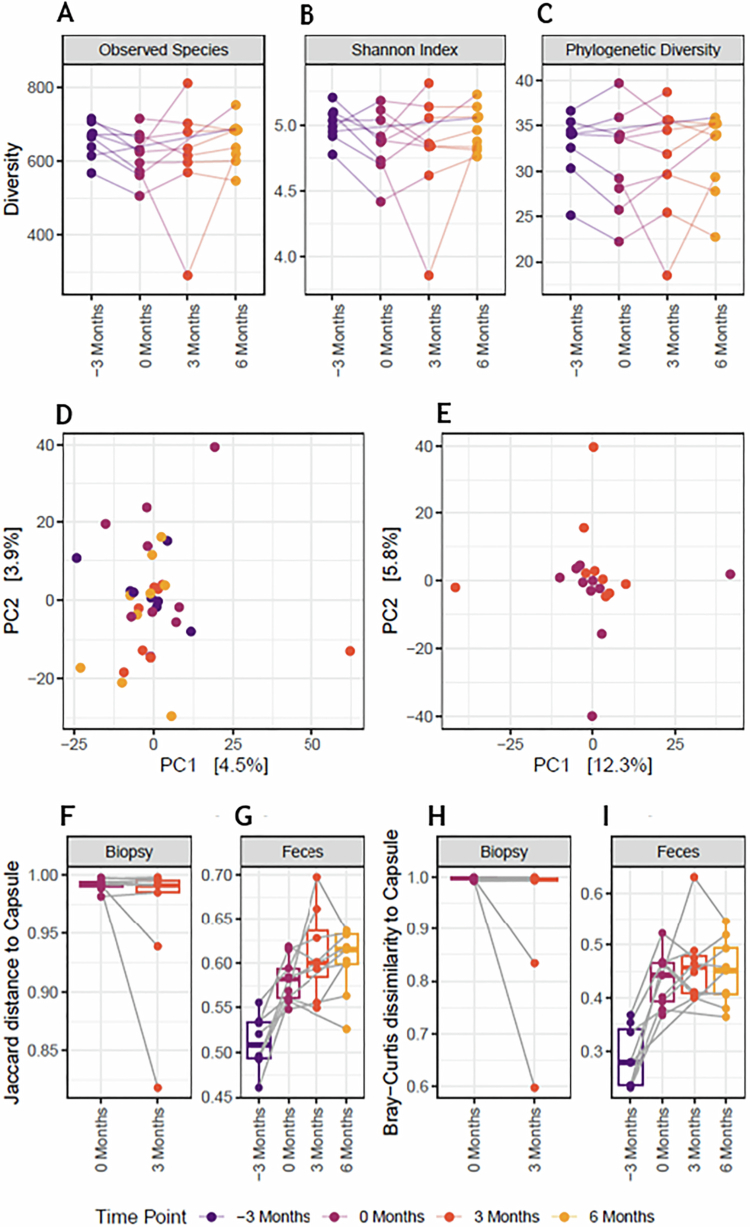
Changes in the small intestinal microbiota and capsule/gut comparison. (a–c) Displays differences in the alpha diversity of the feces samples of timepoint −3 months (dark purple), 0 months (purple), 3 months (orange) and 6 months (yellow). Redundancy analysis of the Bray‒Curtis dissimilarity corrected for inter-individual variance of the feces of all timepoints (d) and the duodenal biopsies at 0 months (purple) and 3 months (orange) (e). Composition of the biopsies (f and h) and feces samples (g and i) and the distance to the corresponding capsule content (f–i). All capsule contents were processed from feces obtained at −3 months. All microbiome analyses were adjusted for multiple testing using Benjamini–Hochberg procedure to control the false discovery rate. A *p*-value of 0.05 was deemed as statistically significant.

Microbial alpha diversity in the duodenal biopsies did not differ before and after treatment or in the fecal samples between all timepoints, as measured with both the Shannon index and phylogenetic diversity (Figure 6a–c). Additionally, there was no difference in microbiome Bray‒Curtis dissimilarity measured by beta diversity or individual species abundance in the biopsies (Figure 6d,e). However, a significant shift (*p* = 0.031, *R*² = 0.19) was observed in the beta diversity of the feces of participants after treatment with a-LFMCs toward the composition of a-LFMCs. When comparing the composition of the a-LFMCs to the duodenal biopsies, we detected a significant decrease in the Jaccard distance of the duodenal microbiome composition and structure towards the capsule composition before and after treatment in some participants, although this difference was not consistent in all participants (Figure 6f–i).

### Duodenal gene expression

**Figure 7.**
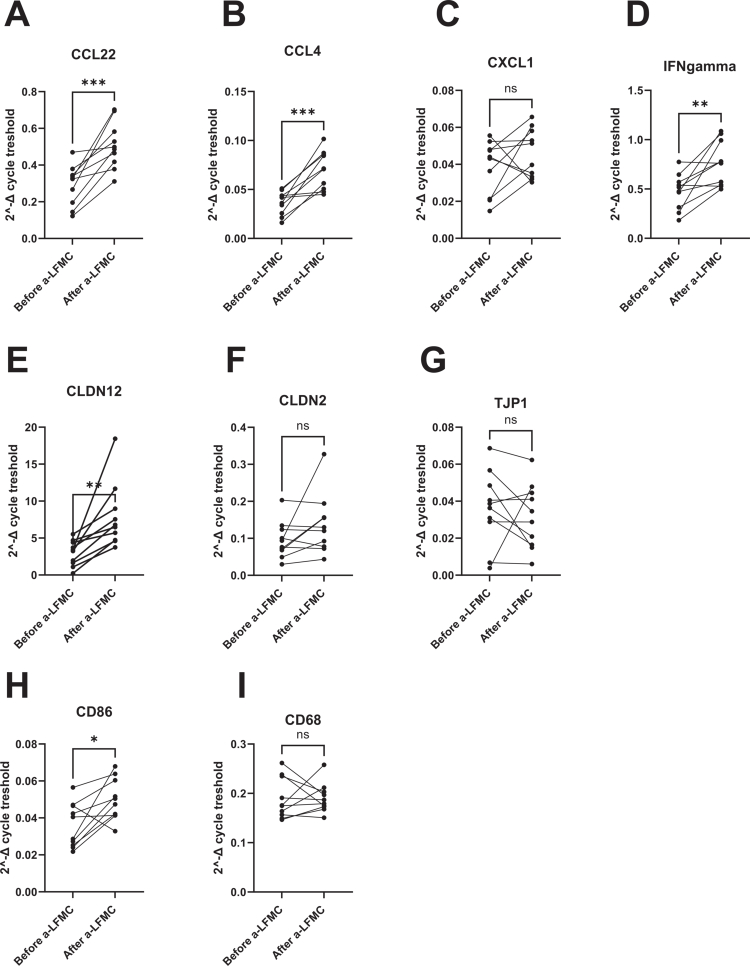
Visualization of gene expression changes in intestinal biopsies pre- and post-a-LFMC treatment. Data represent the mean ± SEM; statistical significance was assessed using paired t-test. Panels A–D show genes involved in immune activation. Panels E–G display genes associated with epithelial barrier function and tight junction integrity. Panels H–I represent immune cell surface markers.

Intestinal biopsies collected before and after a-LFMC treatment revealed significantly greater expression of several key genes associated with immune modulation and barrier function. Specifically, CCL22 expression was significantly increased post-treatment (*p* = 0.0006), as were CLDN12 (*p* = 0.0044), CCL4 (*p* = 0.0005), CD86 (*p* = 0.0142), and IFNγ (*p* = 0.0054) (Figure 7). No significant difference before and after a-LFMC was observed in CXCL1, CLDN2, TJP1, and CD68 expression.

## Discussion

In this open-label, single-arm pilot study, we found that daily ingested a-LFMCs are well-tolerated, safe, and easy to use, resulting in high compliance. During the 3-month capsule ingestion period and the follow-up period, there were no serious adverse events. Furthermore, C-peptide decline appeared to stabilize after the run-in period (−3 to 0 months), with no significant C-peptide decline during the treatment and follow up period. Similarly, both the HbA1c serum concentration and the administered insulin dose significantly increased during run-in but not during the treatment period or during the follow-up. Moreover, we observed no increase in GERD, abdominal pain, indigestion or diarrhea, although some participants reported a transient increase in constipation, further emphasizing its safety. This finding is in line with a recent study where FMT reduced gastrointestinal symptoms in T1D patients.^52^ Finally, we show fecal microbiota viability can remain stable in storage at −80°C for a period up to 2 y, increasing its potential as an easy-to-use treatment. Notably, the viability, as determined by the efficiency of plating of the fecal transplant, decreased after the processing into a-LFMC – this has been observed previously and reflects potential damage to the cells.^53^ However, a significant (19%) fraction of the fecal microbes may also be damaged but could still be viable; hence, further studies on the viability of the a-LFMC preparations by flow cytometry are needed.^54^

Although we are the first to study the safety and efficacy of a-LFMCs in T1D, LFMCs have been studied for multiple diseases, such as recurrent *Clostridioides difficile* infection, obesity and irritable bowel disease.^55^^,^^56^ In this study, we chose daily administration of a-LFMCs; however, no consensus on the best ingestion timing for a-LFMCs has been reached. One study found that a single dose of LFMCs was not inferior to colonoscopy-delivered fecal microbiota in treating people with recurrent *C. difficile* infection.^55^^,^^56^ An RCT in ulcerative colitis patients studied daily LFMC ingestion compared to placebo and found significantly greater remission in the LFMC group after 56 weeks.^57^ This result was similar to another study using daily capsules in the same disease.^58^ Multiple studies in obesity have focused on higher dosages of capsules more closely resembling classic FMT and limiting the dosing to clinical visits,^59^^,^^60^ but found no result in weight reduction. These results suggest that in a chronic illness such as T1D, there is no consensus whether daily ingestion or an intermittent high dose is a more effective mode of delivery for a-LFMCs.

The mechanism by which autologous FMT may halt T1D progression remains elusive.^61^ The use of FMT is still novel in T1D, and to date, only 1 RCT and 2 case-reports document successful treatments. ^23^^,^^62^^,^^63^ One theory on how microbiome perturbations initiate T1D is that duodenal microbiome disruptions trigger immune cell crosstalk. This results in local antimicrobial peptide production, followed by interferon gamma release in the pancreatic beta cells, initiating a diabetogenic CD8 + T-cell response.^20^^,^^64^^,^^65^ Indeed, the previous RCT revealed that when randomized between autologous FMT and allogenic FMT, participants in the autologous group maintained significantly more beta-cell function and had significantly more microbiota strain engraftment in their duodenal biopsies. However, a large meta-analysis on strain engraftment after FMT in different diseases revealed that the clinical results did not always match microbiota strain engraftment.^66^ Although no statistically significant overall shift of the proximal small intestinal gut microbiota was observed, a subset of subjects showed a greater overlap in detected taxa between a-LFMCs content, and samples taken after 3 months of intervention. This suggests that there is some temporal engraftment in the proximal small intestine, although washout might occur rapidly. The evidence for the use of autologous FMT in T1D is mainly derived from consistent findings that coprophagic sterile mice or mice subjected to antibiotics develop T1D at an accelerated pace. Its underlying mechanisms seem to depend on an interaction between innate immune cells and the Th17 response.^61^^,^^67^

Building on our previous work by de Groot et al.,^23^ we selected a panel of candidate genes previously identified to predict the response to FMT with fresh duodenal infusions. Post-FMT, we observed a marked upregulation of CD86, CCL22, and CCL4, collectively indicating increased macrophage infiltration into the duodenum in response to the treatment. This inflammatory signature may reflect a modulation of the local immune environment, potentially shifting away from an autoimmune state. Additionally, the observed increase in CLDN12 expression suggests that FMT may influence tight junction composition and, consequently, intestinal barrier function. To further explore epithelial and immune dynamics, we also assessed CLDN2, TJP1, IFNγ, and CD68. Among these, only IFNγ expression was significantly elevated post-FMT, which is consistent with enhanced Th1-type immune activation, a pathway known to play a complex role in T1D pathogenesis.

To explore potential systemic immunological effects, we assessed a broad panel of 270 cytokines in EDTA plasma using the Nomic platform. No significant changes in plasma cytokine concentrations were observed between the pre- and post-intervention time points (data not shown). This may be attributable to the compartmentalization of immune responses in type 1 diabetes, where the most relevant immunological effects are believed to occur in the pancreas and pancreatic lymph nodes.^68^ As these sites cannot be sampled in living patients, duodenal tissue analyses offer important but indirect mechanistic insights; caution is warranted when extrapolating these local changes to systemic (plasma) immune biomarkers.

Importantly, our study is limited by its single-arm design and reliance solely on gene expression analyses. The absence of detectable changes in plasma cytokines in our cohort, alongside de Groot et al.’s observation of no alterations in circulating immune cells, underscores the challenge of fully elucidating FMT’s mechanisms through non-invasive measures alone. Accordingly, dedicated mechanistic studies employing targeted biopsies and/or animal models will be essential to validate and expand upon these intriguing findings.

### Strengths and limitations

This is primarily a safety study, and although we observed a stabilization of C-peptide AUC decline after a-LFMC treatment, this study had a single-arm design, and the results should be interpreted accordingly.

Our study demonstrated a measurable effect of the intervention in individuals with a median type 1 diabetes (T1D) duration of 2.0 [1.0–3.0] years. We opted for this treatment window for feasibility reasons, and we carefully verified insulin secretion upon inclusion. This is noteworthy, as earlier intervention, especially within weeks of diagnosis, has been shown to offer greater preservation of beta-cell function and improved long-term outcomes.^23^^,^^69^ Nevertheless, our findings suggest that even when intervention is delayed beyond the immediate post-diagnosis period, there is still potential for clinical benefit. Even low levels of endogenous insulin production, as indicated by detectable C-peptide, are associated with clinically important benefits, such as a reduced incidence of severe hypoglycaemia and potentially fewer diabetes-related complications.^70^^,^^71^ Studies have shown that up to 35%–80% of people with established or long-standing T1D retain some residual beta-cell function, and this residual function can contribute to improved glycaemic control and vascular health.^71^ Therefore, interventions targeting the preservation or enhancement of remaining beta-cell activity can yield measurable clinical benefits, even when started after the initial diagnosis.

The participants were all recruited during the period of rapid decline in insulin production that ensues after diagnosis. However, the duration of their disease varies, which has the potential to result in a heterogeneous group, thereby reducing statistical power and leading to a potential underestimation of the treatment effect. However, we already found a clear difference between the run-in and active treatment periods. Nonetheless, to further prove causality, a placebo-controlled trial is needed to confirm our promising findings. The stability of the a-LFMCs was confirmed in the −80 °C freezer for a period of up to 2 y; however, notably, the a-LFMCs were stored in a −20 °C freezer at the participants' own homes during the treatment period. While LFMCs are likely stable at −20 °C, as previously demonstrated by Staley et al.,^72^ direct evidence for our a-LFMCs at this storage temperature was lacking. To address this, we conducted additional stability testing during the rebuttal process, which demonstrated that bacterial viability remained stable for at least three months at −20 °C (Supplementary Figure 1). These findings confirm that storing a-LFMCs at −20 °C for a period of three months is feasible, thereby enhancing the practical applicability of this intervention in real-world settings. However, the stability of a-LFMCs during extended storage at −20 °C has yet to be determined and requires further investigation.

To address our main question of whether the fecal microbiome from a-LFMCs remained detectable in the duodenal biopsies, we used 16S rRNA sequencing because shotgun metagenomics would have overwhelmingly detected human DNA/RNA. Therefore, we also used 16S rRNA sequencing to characterize the capsules and fecal samples to allow for direct comparisons to the duodenal samples. We believe this was the best approach to achieve direct comparisons between these compartments, but we acknowledge that this approach may have missed subtle changes of the microbiome in the fecal samples before and after the treatment. We believe this is an issue for planned follow-up trials, as our current trial is underpowered to link microbial changes to clinical phenotypes.

## Conclusion

a-LFMCs appear to be a safe and potentially promising approach for performing daily FMTs in individuals with type 1 diabetes (T1D). In line with previous studies, autologous FMT may help stabilize endogenous insulin production in individuals diagnosed with T1D between 0.5 and 3.5 years ago. This effect seems to sustain even after a-LFMC administration has stopped. However, as this was a single-arm pilot safety study, these findings should be interpreted as exploratory. Larger, randomized controlled trials are necessary to confirm these observations and determine the efficacy of a-LFMCs in this context.

## Supplementary Material

Supplementary material Supplementary Table 1. Genes and primer sequences used for qPCR.Supplementary Table 2. List of target genes used for proteomics.

## Data Availability

No deidentified individual participant data was shared. The list containing all the microbiota that were examined can be found at the following URL in a public repository: https://github.com/cocofuhri/Metabolites

## Abbreviations

a-LFMCs: Autologous lyophilized fecal microbiota capsules
FMT: Fecal microbiota transplantation
GUTDM1: Abbreviation for the biobank through which data for this manuscript were collected.
HLA: Human leukocyte antigens
MMT: Mixed meal test
T1D: Type 1 diabetes
