# Supplementary material for: Autologous fecal microbiota capsules are safe and potentially preserve beta-cell function in individuals with type 1 diabetes
Source: Gut Microbes. 2025 Oct 5;17(1):2563155. doi: 10.1080/19490976.2025.2563155 (PMC12502823; doi:10.1080/19490976.2025.2563155)
Supplement: Supplementary material — Supplementary Table 1. Genes and primer sequences used for qPCR.Supplementary Table 2. List of target genes used for proteomics. [file KGMI_A_2563155_SM4683.docx]

**Supplementary Table 1.** *Genes and Primer Sequences Used for qPCR*

| **Gene** | **Gene type** | **Primer name** | **Direction** | **Sequence (5’ – 3’)** |
| --- | --- | --- | --- | --- |
| hHPRT1 | Housekeeping | HRPT-F1 | Forward | TGACCTTGATTTATTTTGCATACC |
| hHPRT1 | Housekeeping | HRPT-R1 | Reverse | CGAGCAAGACGTTCAGTCCT |
| hACTB | Housekeeping | huACTBf1 | Forward | CCAACCGCGAGAAGATGA |
| hACTB | Housekeeping | hu ACTB R1 | Reverse | CCAGAGGCGTACAGGGATAG |
| hCCL22 | Target | hCCL22_F2 | Forward | AAGCAACTGAGGCAGGCCC |
| hCCL22 | Target | hCCL22_R2 | Reverse | GGCAGACGGTAACGGACGTA |
| hCD86 | Target | hCD86_F1 | Forward | GAGTGAACAGACCAAGAAAAGAGAA |
| hCD86 | Target | hCD86_R1 | Reverse | AAAAACACGCTGGGCTTCATC |
| hCLDN12 | Target | hCLDN12_F1 | Forward | CAGTTTGCCCTACCCCTCAG |
| hCLDN12 | Target | hCLDN12_R1 | Reverse | CAGTTTGATGTTGGGCACCG |
| hCCL4 | Target | hCCL4_F1 | Forward | CCCAGCCAGCTGTGGTATTC |
| hCCL4 | Target | hCCL4_R1 | Reverse | CCTGGACCCAGGATTCACT |
| hCXCL12 | Target | hCXCL12_F1 | Forward | TGCCCTTCAGATTGTAGCCC |
| hCXCL12 | Target | hCXCL12_R1 | Reverse | TCCACTTTAGCTTCGGGTCA |
| hCD68 | Target | hCD68_F2 | Forward | CCCCAACAAAACCAAGGTCC |
| hCD68 | Target | hCD68_R2 | Reverse | GGA GGT CCT GCA TGA ATC CAA A |
| hCLDN2 | Target | hCLDN2_F1 | Forward | TGGTGCCTGACAGCATGAAA |
| hCLDN2 | Target | hCLDN2_R1 | Reverse | GGGCTTGGTAGGCATCGTAG |
| hTJP1 | Target | hTJP1_F1 | Forward | TTCAGAGTGGGGAAACGTCAAT |
| hTJP1 | Target | hTJP1_R1 | Reverse | GCAACTCGGTCATTTTCCTGTA |

**Supplementary table 2.** List with target genes used for proteomics

| **Nomic Target Name** | **Target Protein** | **Gene Name** | **Uniprot ID** |
| --- | --- | --- | --- |
| Activin A | Inhibin beta A chain | INHBA | P08476 |
| ADAM10 | Disintegrin and metalloproteinase domain-containing protein 10 | ADAM10 | O14672 |
| ADAM17 (TACE) | Disintegrin and metalloproteinase domain-containing protein 17 | ADAM17 | P78536 |
| ADAM28 | Disintegrin and metalloproteinase domain-containing protein 28 | ADAM28 | Q9UKQ2 |
| ADAM9 | Disintegrin and metalloproteinase domain-containing protein 9 | ADAM9 | Q13443 |
| ADAM-TS 1 | A disintegrin and metalloproteinase with thrombospondin motifs 1 | ADAMTS1 | Q9UHI8 |
| ADAM-TS 5 | A disintegrin and metalloproteinase with thrombospondin motifs 5 | ADAMTS5 | Q9UNA0 |
| AITRL (GITR Ligand) | Tumor necrosis factor ligand superfamily member 18 | TNFSF18 | Q9UNG2 |
| Angiopoietin-1 | Angiopoietin-1 | ANGPT1 | Q15389 |
| ANGPTL3 | Angiopoietin-related protein 3 | ANGPTL3 | Q9Y5C1 |
| ANGPTL4 | Angiopoietin-related protein 4 | ANGPTL4 | Q9BY76 |
| ANGPTL7 | Angiopoietin-related protein 7 | ANGPTL7 | O43827 |
| APRIL | Tumor necrosis factor ligand superfamily member 13 | TNFSF13 | O75888 |
| BAFF | Tumor necrosis factor ligand superfamily member 13B | TNFSF13B | Q9Y275 |
| BCMA (TNFRSF17) | Tumor necrosis factor receptor superfamily member 17 | TNFRSF17 | Q02223 |
| BDNF | Brain-derived neurotrophic factor | BDNF | P23560 |
| BMP2 | Bone morphogenetic protein 2 | BMP2 | P12643 |
| BMP3 | Bone morphogenetic protein 3 | BMP3 | P12645 |
| BMP4 | Bone morphogenetic protein 4 | BMP4 | P12644 |
| BMP6 | Bone morphogenetic protein 6 | BMP6 | P22004 |
| BMP7 | Bone morphogenetic protein 7 | BMP7 | P18075 |
| BMP9 | Growth/differentiation factor 2 | GDF2 | Q9UK05 |
| Brevican | Brevican core protein | BCAN | Q96GW7 |
| C5/C5a | Complement C5 | C5 | P01031 |
| Calbindin | Calbindin | CALB1 | P05937 |
| CCL1 | C-C motif chemokine 1 | CCL1 | P22362 |
| CCL11 | Eotaxin | CCL11 | P51671 |
| CCL13 | C-C motif chemokine 13 | CCL13 | Q99616 |
| CCL14 | C-C motif chemokine 14 | CCL14 | Q16627 |
| CCL15 | C-C motif chemokine 15 | CCL15 | Q16663 |
| CCL16 | C-C motif chemokine 16 | CCL16 | O15467 |
| CCL17 | C-C motif chemokine 17 | CCL17 | Q92583 |
| CCL18 | C-C motif chemokine 18 | CCL18 | P55774 |
| CCL19 | C-C motif chemokine 19 | CCL19 | Q99731 |
| CCL2 | C-C motif chemokine 2 | CCL2 | P13500 |
| CCL20 | C-C motif chemokine 20 | CCL20 | P78556 |
| CCL21 | C-C motif chemokine 21 | CCL21 | O00585 |
| CCL22 | C-C motif chemokine 22 | CCL22 | O00626 |
| CCL23 | C-C motif chemokine 23 | CCL23 | P55773 |
| CCL24 | C-C motif chemokine 24 | CCL24 | O00175 |
| CCL25 | C-C motif chemokine 25 | CCL25 | O15444 |
| CCL26 | C-C motif chemokine 26 | CCL26 | Q9Y258 |
| CCL27 | C-C motif chemokine 27 | CCL27 | Q9Y4X3 |
| CCL28 | C-C motif chemokine 28 | CCL28 | Q9NRJ3 |
| CCL3 | C-C motif chemokine 3 | CCL3 | P10147 |
| CCL4 | C-C motif chemokine 4 | CCL4 | P13236 |
| CCL5 | C-C motif chemokine 5 | CCL5 | P13501 |
| CCL7 | C-C motif chemokine 7 | CCL7 | P80098 |
| CD14 | Monocyte differentiation antigen CD14 | CD14 | P08571 |
| CD163 | Scavenger receptor cysteine-rich type 1 protein M130 | CD163 | Q86VB7 |
| CD209 | CD209 antigen | CD209 | Q9NNX6 |
| CD27L | CD70 antigen | CD70 | P32970 |
| CD30 | Tumor necrosis factor receptor superfamily member 8 | TNFRSF8 | P28908 |
| CD40L | CD40 ligand | CD40LG | P29965 |
| CD80 (B7-1) | T-lymphocyte activation antigen CD80 | CD80 | P33681 |
| CD86 (B7-2) | T-lymphocyte activation antigen CD86 | CD86 | P42081 |
| CHI3L1 | Chitinase-3-like protein 1 | CHI3L1 | P36222 |
| CLC | Cardiotrophin-like cytokine factor 1 | CLCF1 | Q9UBD9 |
| CNTF | Ciliary neurotrophic factor | CNTF | P26441 |
| CX3CL1 | Fractalkine | CX3CL1 | P78423 |
| CXCL1 | Growth-regulated alpha protein | CXCL1 | P09341 |
| CXCL10 | C-X-C motif chemokine 10 | CXCL10 | P02778 |
| CXCL11 | C-X-C motif chemokine 11 | CXCL11 | O14625 |
| CXCL12 (alpha),CXCL12 (beta) | Stromal cell-derived factor 1 | CXCL12 | P48061 |
| CXCL12 (alpha),CXCL12 (beta) | Stromal cell-derived factor 1 | CXCL12 | P48061 |
| CXCL13 | C-X-C motif chemokine 13 | CXCL13 | O43927 |
| CXCL14 | C-X-C motif chemokine 14 | CXCL14 | O95715 |
| CXCL16 | C-X-C motif chemokine 16 | CXCL16 | Q9H2A7 |
| CXCL17 | C-X-C motif chemokine 17 | CXCL17 | Q6UXB2 |
| CXCL3 | C-X-C motif chemokine 3 | CXCL3 | P19876 |
| CXCL4 | Platelet factor 4 | PF4 | P02776 |
| CXCL5 | C-X-C motif chemokine 5 | CXCL5 | P42830 |
| CXCL6 | C-X-C motif chemokine 6 | CXCL6 | P80162 |
| CXCL7 | Platelet basic protein | PPBP | P02775 |
| CXCL9 | C-X-C motif chemokine 9 | CXCL9 | Q07325 |
| Cytochrome C | Cytochrome c | CYCS | P99999 |
| Decorin | Decorin | DCN | P07585 |
| DPP4 (CD26) | Dipeptidyl peptidase 4 | DPP4 | P27487 |
| DR3 | Tumor necrosis factor receptor superfamily member 25 | TNFRSF25 | Q93038 |
| DR6 | Tumor necrosis factor receptor superfamily member 21 | TNFRSF21 | O75509 |
| ECM1 | Extracellular matrix protein 1 | ECM1 | Q16610 |
| EGF | Pro-epidermal growth factor | EGF | P01133 |
| EGFR | Epidermal growth factor receptor | EGFR | P00533 |
| ELA2 | Neutrophil elastase | ELANE | P08246 |
| EMMPRIN | Basigin | BSG | P35613 |
| Endoglin (CD105) | Endoglin | ENG | P17813 |
| E-Selectin (CD62E) | E-selectin | SELE | P16581 |
| FAS | Tumor necrosis factor receptor superfamily member 6 | FAS | P25445 |
| FAS-L | Tumor necrosis factor ligand superfamily member 6 | FASLG | P48023 |
| FASN | Fatty acid synthase | FASN | P49327 |
| Fetuin-B | Fetuin-B | FETUB | Q9UGM5 |
| FGF-1 | Fibroblast growth factor 1 | FGF1 | P05230 |
| FGF-19 | Fibroblast growth factor 19 | FGF19 | O95750 |
| FGF-2 | Fibroblast growth factor 2 | FGF2 | P09038 |
| FGF-21 | Fibroblast growth factor 21 | FGF21 | Q9NSA1 |
| FGF-4 | Fibroblast growth factor 4 | FGF4 | P08620 |
| FGF-6 | Fibroblast growth factor 6 | FGF6 | P10767 |
| FGF-7 (KGF) | Fibroblast growth factor 7 | FGF7 | P21781 |
| FGF-9 | Fibroblast growth factor 9 | FGF9 | P31371 |
| FGFR3 (IIIc) | Fibroblast growth factor receptor 3 | FGFR3 | P22607 |
| Fibulin 3 | EGF-containing fibulin-like extracellular matrix protein 1 | EFEMP1 | Q12805 |
| Ficolin-2 | Ficolin-2 | FCN2 | Q15485 |
| FLRG (FSTL3) | Follistatin-related protein 3 | FSTL3 | O95633 |
| Flt-3 | Receptor-type tyrosine-protein kinase FLT3 | FLT3 | P36888 |
| Flt-3 Ligand | Fms-related tyrosine kinase 3 ligand | FLT3LG | P49771 |
| Galectin-1 | Galectin-1 | LGALS1 | P09382 |
| Galectin-3 | Galectin-3 | LGALS3 | P17931 |
| Galectin-9 | Galectin-9 | LGALS9 | O00182 |
| GAS6 | Growth arrest-specific protein 6 | GAS6 | Q14393 |
| G-CSF | Granulocyte colony-stimulating factor | CSF3 | P09919 |
| GDF-11 (BMP-11) | Growth/differentiation factor 11 | GDF11 | O95390 |
| GDF-15 (MIC-1) | Growth/differentiation factor 15 | GDF15 | Q99988 |
| GDNF | Glial cell line-derived neurotrophic factor | GDNF | P39905 |
| GM-CSF | Granulocyte-macrophage colony-stimulating factor | CSF2 | P04141 |
| Granzyme B | Granzyme B | GZMB | P10144 |
| HGF | Hepatocyte growth factor | HGF | P14210 |
| HGF R (c-MET) | Hepatocyte growth factor receptor | MET | P08581 |
| HVEM | Tumor necrosis factor receptor superfamily member 14 | TNFRSF14 | Q92956 |
| ICAM-1 | Intercellular adhesion molecule 1 | ICAM1 | P05362 |
| ICAM-2 | Intercellular adhesion molecule 2 | ICAM2 | P13598 |
| ICOSL (B7-H2) | ICOS ligand | ICOSLG | O75144 |
| IFN alpha 2 (alpha 2b) | Interferon alpha-2 | IFNA2 | P01563 |
| IFN beta | Interferon beta | IFNB1 | P01574 |
| IFN gamma | Interferon gamma | IFNG | P01579 |
| IFN-epsilon | Interferon epsilon | IFNE | Q86WN2 |
| IGF-1 | Insulin-like growth factor I | IGF1 | P05019 |
| IGFBP3 | Insulin-like growth factor-binding protein 3 | IGFBP3 | P17936 |
| IGFBP6 | Insulin-like growth factor-binding protein 6 | IGFBP6 | P24592 |
| IL-10 | Interleukin-10 | IL10 | P22301 |
| IL-11 | Interleukin-11 | IL11 | P20809 |
| IL-12 p35,IL-12 p70 | Interleukin-12 subunit alpha | IL12A | P29459 |
| IL-12 p40 | Interleukin-12 subunit beta | IL12B | P29460 |
| IL-12 RB1 | Interleukin-12 receptor subunit beta-1 | IL12RB1 | P42701 |
| IL-13 | Interleukin-13 | IL13 | P35225 |
| IL13RA1 | Interleukin-13 receptor subunit alpha-1 | IL13RA1 | P78552 |
| IL-15/IL-15R alpha complex,IL-15 | Interleukin-15 | IL15 | P40933 |
| IL-16 | Pro-interleukin-16 | IL16 | Q14005 |
| IL-17A | Interleukin-17A | IL17A | Q16552 |
| IL-17B | Interleukin-17B | IL17B | Q9UHF5 |
| IL-17C | Interleukin-17C | IL17C | Q9P0M4 |
| IL-17D | Interleukin-17D | IL17D | Q8TAD2 |
| IL-17E (IL-25) | Interleukin-25 | IL25 | Q9H293 |
| IL-17F | Interleukin-17F | IL17F | Q96PD4 |
| IL-18 | Interleukin-18 | IL18 | Q14116 |
| IL-1RAP | Interleukin-1 receptor accessory protein | IL1RAP | Q9NPH3 |
| IL-2 | Interleukin-2 | IL2 | P60568 |
| IL-21 | Interleukin-21 | IL21 | Q9HBE4 |
| IL-22 | Interleukin-22 | IL22 | Q9GZX6 |
| IL-22 BP | Interleukin-22 receptor subunit alpha-2 | IL22RA2 | Q969J5 |
| IL-23 | Interleukin-23 subunit alpha | IL23A | Q9NPF7 |
| IL-24 | Interleukin-24 | IL24 | Q13007 |
| IL-27 | Interleukin-27 subunit alpha | IL27 | Q8NEV9 |
| IL-28A | Interferon lambda-2 | IFNL2 | Q8IZJ0 |
| IL-29 | Interferon lambda-1 | IFNL1 | Q8IU54 |
| IL-3 | Interleukin-3 | IL3 | P08700 |
| IL-31 | Interleukin-31 | IL31 | Q6EBC2 |
| IL-32 (alpha) | Interleukin-32 | IL32 | P24001 |
| IL-33 | Interleukin-33 | IL33 | O95760 |
| IL-34 | Interleukin-34 | IL34 | Q6ZMJ4 |
| IL-35 | Interleukin-27 subunit beta | EBI3 | Q14213 |
| IL-36a | Interleukin-36 alpha | IL36A | Q9UHA7 |
| IL-4 | Interleukin-4 | IL4 | P05112 |
| IL-4 R | Interleukin-4 receptor subunit alpha | IL4R | P24394 |
| IL-5 | Interleukin-5 | IL5 | P05113 |
| IL-6 | Interleukin-6 | IL6 | P05231 |
| IL-7 | Interleukin-7 | IL7 | P13232 |
| IL-8 | Interleukin-8 | CXCL8 | P10145 |
| IL-9 | Interleukin-9 | IL9 | P15248 |
| Kit (CD117) | Mast/stem cell growth factor receptor Kit | KIT | P10721 |
| Leptin | Leptin | LEP | P41159 |
| LIF | Leukemia inhibitory factor | LIF | P15018 |
| LOX1 (OLR1) | Oxidized low-density lipoprotein receptor 1 | OLR1 | P78380 |
| L-selectin (CD62L) | L-selectin | SELL | P14151 |
| MBL2 (MBP1) | Mannose-binding protein C | MBL2 | P11226 |
| M-CSF | Macrophage colony-stimulating factor 1 | CSF1 | P09603 |
| M-CSF R (CD115) | Macrophage colony-stimulating factor 1 receptor | CSF1R | P07333 |
| MIF | Macrophage migration inhibitory factor | MIF | P14174 |
| MMP-1 | Interstitial collagenase | MMP1 | P03956 |
| MMP-10 | Stromelysin-2 | MMP10 | P09238 |
| MMP-12 | Macrophage metalloelastase | MMP12 | P39900 |
| MMP-13 | Collagenase 3 | MMP13 | P45452 |
| MMP-2 | 72 kDa type IV collagenase | MMP2 | P08253 |
| MMP-3 | Stromelysin-1 | MMP3 | P08254 |
| MMP-7 | Matrilysin | MMP7 | P09237 |
| MMP-9 | Matrix metalloproteinase-9 | MMP9 | P14780 |
| NF-L | Neurofilament light polypeptide | NEFL | P07196 |
| NGAL (lipocalin-2) | Neutrophil gelatinase-associated lipocalin | LCN2 | P80188 |
| NGF beta | Beta-nerve growth factor | NGF | P01138 |
| NRG1 beta 1 | Pro-neuregulin-1, membrane-bound isoform | NRG1 | Q02297 |
| Oncostatin M (OSM) | Oncostatin-M | OSM | P13725 |
| Osteoactivin (GPNMB) | Transmembrane glycoprotein NMB | GPNMB | Q14956 |
| Osteopontin (OPN) | Osteopontin | SPP1 | P10451 |
| PCOLCE (PCPE1) | Procollagen C-endopeptidase enhancer 1 | PCOLCE | Q15113 |
| PCSK9 | Proprotein convertase subtilisin/kexin type 9 | PCSK9 | Q8NBP7 |
| PD-1 | Programmed cell death protein 1 | PDCD1 | Q15116 |
| PDGF-BB | Platelet-derived growth factor subunit B | PDGFB | P01127 |
| PD-L1 (B7-H1) | Programmed cell death 1 ligand 1 | CD274 | Q9NZQ7 |
| PD-L2 | Programmed cell death 1 ligand 2 | PDCD1LG2 | Q9BQ51 |
| PGLYRP1 (PGRP-S) | Peptidoglycan recognition protein 1 | PGLYRP1 | O75594 |
| PlGF,PLGF | Placenta growth factor | PGF | P49763 |
| Prolargin | Prolargin | PRELP | P51888 |
| PTX3 (Pentraxin 3) | Pentraxin-related protein PTX3 | PTX3 | P26022 |
| Resistin | Resistin | RETN | Q9HD89 |
| SAA | Serum amyloid A-1 protein | SAA1 | P0DJI8 |
| SCF | Kit ligand | KITLG | P21583 |
| SLAMF1 (CD150) | Signaling lymphocytic activation molecule | SLAMF1 | Q13291 |
| TGF-beta 1 (total),TGF-beta 1 (LAP domain in precursor) | Transforming growth factor beta-1 proprotein | TGFB1 | P01137 |
| TGF-beta 2 | Transforming growth factor beta-2 proprotein | TGFB2 | P61812 |
| TGF-beta 3 | Transforming growth factor beta-3 proprotein | TGFB3 | P10600 |
| TGFBR3 | Transforming growth factor beta receptor type 3 | TGFBR3 | Q03167 |
| Thrombomodulin | Thrombomodulin | THBD | P07204 |
| Thrombospondin-1 | Thrombospondin-1 | THBS1 | P07996 |
| Tie-2 | Angiopoietin-1 receptor | TEK | Q02763 |
| TIMP1 | Metalloproteinase inhibitor 1 | TIMP1 | P01033 |
| TIMP2 | Metalloproteinase inhibitor 2 | TIMP2 | P16035 |
| TIMP4 | Metalloproteinase inhibitor 4 | TIMP4 | Q99727 |
| Tissue Factor (TF) | Tissue factor | F3 | P13726 |
| TLR2 | Toll-like receptor 2 | TLR2 | O60603 |
| TNF alpha | Tumor necrosis factor | TNF | P01375 |
| TNF beta | Lymphotoxin-alpha | LTA | P01374 |
| TNF RI | Tumor necrosis factor receptor superfamily member 1A | TNFRSF1A | P19438 |
| TNF RII | Tumor necrosis factor receptor superfamily member 1B | TNFRSF1B | P20333 |
| TNF RIII (Lymphotoxin Beta R) | Tumor necrosis factor receptor superfamily member 3 | LTBR | P36941 |
| TNFSF9 | Tumor necrosis factor ligand superfamily member 9 | TNFSF9 | P41273 |
| TPO (Thrombopoietin) | Thrombopoietin | THPO | P40225 |
| TRAIL | Tumor necrosis factor ligand superfamily member 10 | TNFSF10 | P50591 |
| TREM1 | Triggering receptor expressed on myeloid cells 1 | TREM1 | Q9NP99 |
| TSLP | Thymic stromal lymphopoietin | TSLP | Q969D9 |
| TWEAK | Tumor necrosis factor ligand superfamily member 12 | TNFSF12 | O43508 |
| TWEAK R (CD266) | Tumor necrosis factor receptor superfamily member 12A | TNFRSF12A | Q9NP84 |
| uPA | Urokinase-type plasminogen activator | PLAU | P00749 |
| uPA R | Urokinase plasminogen activator surface receptor | PLAUR | Q03405 |
| VCAM-1 | Vascular cell adhesion protein 1 | VCAM1 | P19320 |
| VEGF-A (165) | Vascular endothelial growth factor A, long form | VEGFA | P15692 |
| VEGF-C | Vascular endothelial growth factor C | VEGFC | P49767 |
| VEGF-D | Vascular endothelial growth factor D | VEGFD | O43915 |
| VEGFR-1 | Vascular endothelial growth factor receptor 1 | FLT1 | P17948 |
| VISTA | V-type immunoglobulin domain-containing suppressor of T-cell activation | VSIR | Q9H7M9 |
| WIF-1 | Wnt inhibitory factor 1 | WIF1 | Q9Y5W5 |
| WISP-1 (CCN4) | CCN family member 4 | CCN4 | O95388 |
| Wnt-3a | Protein Wnt-3a | WNT3A | P56704 |
| Wnt-5a | Protein Wnt-5a | WNT5A | P41221 |
| XCL1 (Lymphotactin) | Lymphotactin | XCL1 | P47992 |

**Supplementary Table 3.** *The presence of gastrointestinal symptoms between al timepoints. Mixed meal tolerance test 1 (MMT1) was timepoint -3 months, Mixed meal tolerance test 2 (MMT2) was timepoint 0 months, Mixed meal tolerance test 3 (MMT3) was timepoint 3 months, Mixed meal tolerance test 4 (MMT4) was timepoint 6 months. GI-symptoms were divided in yes or no based on the gastrointestinal symptoms rating scale (GSRS), individuals with a score of < 3 were classified as no clinically relevant GI-symptoms (No) and a score of ≥ 3 classified as clinically relevant (Yes). All syndromes listed were abdominal pain syndrome, congestion syndrome, indigestion syndrome, diarrhoea syndrome and gastro-oesophageal reflux disease (GERD) and an ANOVA was used to test for association.*

|  | MMT1 | MMT2 | MMT3 | MMT4 | p |
| --- | --- | --- | --- | --- | --- |
| n | 10 | 10 | 10 | 10 |  |
| Abdominal Pain - Yes (%) | 1 (10.0) | 2 (20.0) | 1 (10.0) | 0 (0.0) | 0.528 |
| Diarrhoea - Yes (%) | 0 (0.0) | 1 (10.0) | 0 (0.0) | 3 (30.0) | 0.083 |
| Constipation- Yes (%) | 0 (0.0) | 0 (0.0) | 3 (30.0) | 0 (0.0) | 0.021 |
| Indigestion -Yes (%) | 1 (10.0) | 1 (10.0) | 1 (10.0) | 1 (10.0) | 1.000 |
| GERD - Yes (%) | 0 (0.0) | 0 (0.0) | 0 (0.0) | 0 (0.0) | NA |
| Any GI Symptoms -Yes (%) | 1 (10.0) | 3 (30.0) | 4 (40.0) | 3 (30.0) | 0.497 |

**Supplementary Table 4.** *Missing values per variable (n=10)*

|  | MMT1 |  | MMT2 |  | MMT3 |  | MMT4 |  |
| --- | --- | --- | --- | --- | --- | --- | --- | --- |
|  | Missing values (n) | Missing values (%) | Missing values (n) | Missing values (%) | Missing values (n) | Missing values (%) | Missing values (n) | Missing values (%) |
| Characteristic | 0 | 0 | 0 | 0 | 0 | 0 | 0 | 0 |
| Participants (n) | 0 | 0 | 0 | 0 | 0 | 0 | 0 | 0 |
| Male sex (%) | 0 | 0 | 0 | 0 | 0 | 0 | 0 | 0 |
| Age (years) | 0 | 0 | 0 | 0 | 0 | 0 | 0 | 0 |
| BMI (kg/m2) | 0 | 0 | 0 | 0 | 0 | 0 | 0 | 0 |
| Duration of T1D (years) | 0 | 0 | 0 | 0 | 0 | 0 | 0 | 0 |
| Age of Onset (%) | 0 | 0 | 0 | 0 | 0 | 0 | 0 | 0 |
| Time in range (%) | 0 | 0 | 1 | 10 | 1 | 10 | 1 | 10 |
| Time above range (%) | 0 | 0 | 1 | 10 | 1 | 10 | 1 | 10 |
| Time below range (%) | 0 | 0 | 1 | 10 | 1 | 10 | 1 | 10 |
| Glucose CV (%) | 1 | 10 | 2 | 20 | 2 | 20 | 2 | 20 |
| Fasting C-peptide (%) | 0 | 0 | 1 | 10 | 0 | 0 | 0 | 0 |
| HbA1c (mmol/mmol) | 0 | 0 | 1 | 10 | 0 | 0 | 0 | 0 |
| Fasting Glucose (mmol/L) | 0 | 0 | 0 | 0 | 0 | 0 | 0 | 0 |
| Daily insulin dose (U/day) | 0 | 0 | 0 | 0 | 0 | 0 | 0 | 0 |
| Total cholesterol (mmol/L) | 0 | 0 | 0 | 0 | 0 | 0 | 0 | 0 |
| LDL (mmol/L) | 0 | 0 | 0 | 0 | 0 | 0 | 0 | 0 |
| HDL (mmol/L) | 0 | 0 | 0 | 0 | 0 | 0 | 0 | 0 |
| HLA | 2 | 20 | - | - | - | - | - | - |
| Total energy intake (kJ/day) | 0 | 0 | 1 | 10 | 1 | 10 | 1 | 10 |
| Fibre intake (g/day) | 0 | 0 | 1 | 10 | 1 | 10 | 1 | 10 |
| Protein intake (g/day) | 0 | 0 | 1 | 10 | 1 | 10 | 1 | 10 |
| Carbohydrate intake (g/day) | 0 | 0 | 1 | 10 | 1 | 10 | 1 | 10 |
| Fat intake (g/day) | 0 | 0 | 1 | 10 | 1 | 10 | 1 | 10 |
| Sugar intake (g/day) | 1 | 10 | 1 | 10 | 1 | 10 | 1 | 10 |

**Supplementary Figure 1.** *LFMC stability of both anaerobic and aerobic bacteria at room temperature (RT), 4 ℃, -20 ℃ and -80 ℃ during 3 months of storage.*
